# Use of Complementary and Alternative Medicine by Patients with Irritable Bowel Syndrome According to the Roma IV Criteria: A Single-Center Italian Survey

**DOI:** 10.3390/medicina55020046

**Published:** 2019-02-13

**Authors:** Tiziana Larussa, Marianna Rossi, Evelina Suraci, Raffaella Marasco, Maria Imeneo, Ludovico Abenavoli, Francesco Luzza

**Affiliations:** Department of Health Sciences, University of Catanzaro “Magna Graecia”, Viale Europa, 88100 Catanzaro, Italy; tiziana.larussa@gmail.com (T.L.); marianna.rossi903@virgilio.it (M.R.); e.suraci@libero.it (E.S.); raffaellamarasco1@gmail.com (R.M.); graziaimeneo@hotmail.it (M.I.); l.abenavoli@unicz.it (L.A.)

**Keywords:** irritable bowel syndrome, complementary and alternative medicine, patient-centered care, Rome IV criteria, nutritional supplements, health behaviors, public health

## Abstract

*Aim:* This study was conducted to evaluate the impact of complementary and alternative medicine (CAM) in patients with irritable bowel syndrome (IBS) as assessed by the Rome IV criteria. *Methods:* Consecutive patients referring for IBS were re-evaluated according to the Rome IV criteria. Demographic features and characteristics potentially associated with the use of CAM were collected. A validated, self-administered, survey questionnaire dealing with CAM and patients’ level of knowledge, motivation, perception, and information seeking-behavior toward the use of CAM was analyzed. Multivariate logistic regression analysis was performed in order to identify predictors of CAM use among participants. *Results:* Among 156 patients claiming IBS, 137 (88%) met the Rome IV criteria, and 62 of them (45%) were CAM users. Biologically based therapy was the most chosen CAM (78%). Significant risk factors (adjusted odds ratio, 95% confidence interval) for the use of CAM were female gender (7.22, 2.31–22.51), a higher BMI (1.16, 1.02–1.33), and a good knowledge of CAM (4.46, 1.73–11.45), while having children was a protective factor (0.25, 0.07–0.95). Only 19% of patients used CAM due to medical advice and over half (51%) thought it was a “more natural” approach. Although a minority of patients (16%) had full satisfaction from CAM, 81% of users would repeat the CAM experience for their IBS symptoms. *Conclusions*: The widespread use of CAM in IBS, the patients’ belief in its safety, and their willingness to re-use it suggest that knowledge of health-care providers and patient education should be improved.

## 1. Introduction

Irritable bowel syndrome (IBS) is the most common functional gastrointestinal disorder, affecting 7–15% of the general population [1]. It is a condition characterized by a combination of chronic abdominal pain associated with a change in the frequency or form of stool [2]. Clinical characterization of IBS remains difficult due to the heterogeneous phenotypes, as well as the etiology, the pathophysiological mechanisms of which are still under investigation [3,4]. Currently, it is recognized that alterations in the gut microbiome and gut immune function, changes in intestinal motility and permeability, visceral hypersensitivity, brain–gut interactions, psychosocial status, and food components are implicated, to some degree, in the development of IBS [5]. At present, treatments are targeted toward these plausible mechanisms, but benefits from drug therapy are limited and the negative impact of IBS on quality of life involves a significant use burden on healthcare resources, with high direct and indirect costs [6].

The recent Rome IV criteria eliminated the term non-specific “discomfort” and considered the IBS with predominant diarrhea (IBS-D), IBS with predominant constipation (IBS-C), and IBS with mixed bowel habits (IBS-M) as a continuous disorder [7]. 

Complementary and alternative medicine (CAM) comprises a broad set of medical products and practices that generally do not account for conventional medicine and are classified and updated according to the annual survey performed by the National Health Interview Survey [8]. These natural remedies are emerging in gastroenterology, offering promising options for pathologies such as inflammatory bowel disease (IBD) [9,10]. CAM is widely employed in the treatment of IBS and up to 50% of patients declared that they used some form of CAM for their gastrointestinal symptoms, ranging from biologically active compounds to mind–body interventions [11,12]. Despite the wide dissemination of the therapeutic CAM approach, poor epidemiological and clinical data are available, especially from within the Italian population. More investigation on the use of CAM for IBS is needed, in order to provide clinicians with a proper handling of this growing phenomenon.

The aim of this study was to assess the prevalence, risk factors, and motivations for CAM use in IBS patients, according to the Rome IV criteria, through a monocentric survey.

## 2. Materials and Methods

### 2.1. Patients

From June 2017 to September 2017, a monocentric survey was conducted at the Digestive Pathophysiology Unit of the University Hospital in Catanzaro, in Southern Italy, to investigate the use of CAM in subjects with IBS. The aim of the survey was to collect data on the prevalence and types of CAM practiced, and the characteristics of their users. Patients aged ≥18 years, who were consecutively admitted to the outpatient clinic with a previous diagnosis of IBS, were re-evaluated for this condition according to the Roma IV criteria (Table 1) [13]. In those patients where there were features that could be a concern for their inclusion in the study (such as an onset of symptoms after 50 years of age, rectal bleeding, unexplained weight loss, family history of organic gastrointestinal diseases, and unexplained iron-deficiency anemia), selected tests were performed as appropriate to exclude organic diseases that can mimic IBS (e.g., complete blood cell count, C-reactive protein or fecal calprotectin, serologic testing for celiac disease, and colonoscopy). Each patient was interviewed by the trained clinical team, and information concerning demographic characteristics, lifestyle, type of work, level of education, previous relevant medical history, current co-morbidity, and the type of medications used was collated. In addition, a careful family history was carried out in order to assess the presence of diseases with a strong emotional impact such as cancer. Self-reported height and weight were converted to metric units for the calculation of body mass index (BMI), and patients were classified as underweight, normal weight, overweight, or obese, accordingly. Exclusion criteria were the presence of a gastrointestinal condition other than IBS (e.g., celiac disease, IBD, and diverticular disease) when it could be considered the main disease responsible for the current symptoms, and previous surgical gastrointestinal interventions to determine alterations of digestive functions.

### 2.2. Intervention

If a patient was classified as IBS-suffering according to the Rome IV criteria, he/she was asked to fill a questionnaire about CAM therapies for IBS. Participants were informed that the aim of the study was to assess the extent to which patients with IBS experienced and benefit from CAM therapies. Patients were asked to complete the survey in a waiting room and to return the form before leaving the hospital. A trained clinician was available if patients raised any questions or had any doubts, or encountered difficulties in completing the survey. The questionnaire did not contain identifiable data but there was a code for analyzing purpose, and patients were assured that the data would remain confidential.

### 2.3. Questionnaire

The questionnaire was a self-administered one, designed and constructed after a thorough literature review [14,15,16] to investigate the use of CAM in subjects with functional gastrointestinal diseases, and took around 10 min to complete (Supplementary Materials). Before starting the study, the survey was administered to 20 volunteers, to evaluate its feasibility and clarity. The content validity of the questionnaire was assessed with the help of interviews and comparison between the two methods (κ = 0.70). Seven items addressed the attitudes, preferences, and satisfaction outcomes of patients regarding CAM therapies, and their experience with traditional medicine options, over the last 12 months. CAM therapies listed included alternative medical systems (e.g., homeopathy, Chinese medicine, and Ayurvedic medicine), mind–body interventions (e.g., meditation, prayer, mental training, and art therapy), biologically based therapies (e.g., herbs, foods and nutritional supplements), manipulation therapies (e.g., chiropractic and massage), and energy therapies (e.g., healing touch and bioelectromagnetically based therapies), according to the US National Institutes of Health [8]. Furthermore, patients were invited to detail who recommended the use of CAM therapies for their gastrointestinal symptoms. 

### 2.4. Statistics

As the assessment of the normality of data is an underlying assumption in choosing parametric or non-parametric tests, the Kolmogorov–Smirnov test of normality was used to analyze the data distribution. Continuous data were expressed as a mean plus SD when normally distributed and as a median with a range if not. The socio-demographic and clinical characteristics were compared with a *T*-test (for normally distributed data) or a Mann–Whitney U-test (for not normally distributed data) for continuous variables and chi-square test for categorical variables. Binary logistic regression analysis was performed in order to identify predictors of CAM use among participants. The odds ratio (OR) of treating IBS with CAM therapy, given the presence of a particular variable, was used as a measure of association and adjusted for the effect of confounding variables. The agreement between the questionnaire and reports from the medical interview, obtained during the pre-test in volunteers, was evaluated by means of the κ statistic, which is a measure of the agreement between two observers or tests. Values range from 0 and 1, where 0 indicates an agreement expected on the basis of chance alone and 1 indicating a complete (100%) agreement. Statistical analysis was performed using the PASW statistic 18.0 software (IBM SPSS Statistics, Chicago, IL, USA). A *p*-value of less than 0.05 was considered statistically significant.

### 2.5. Ethical Considerations

The study was carried out according to the Declaration of Helsinki. The patients received oral and written information about the study. All participants were informed that participation was voluntarily and that they could withdraw at any time without consequences. The study protocol was approved by the local research Ethical Committee “Magna Graecia University” (n. 182/17), and written informed consent was obtained from all the participants.

## 3. Results

### 3.1. Demographic and Clinical Characteristics of Study Population

Of the 156 patients who were consecutively admitted to the outpatient clinic with a previous diagnosis of IBS, 137 (88%) met the Rome IV criteria and accepted to fill the survey form. No patient had gastrointestinal cancer or history of IBD, nor had undergone intestinal surgery. One female patient suffered from celiac disease but was complying with a gluten-free diet, and all serological and histological tests performed at site indicated that the disease was under control. All the participants were native local residents. Through the clinical interview, no special diet regimens (e.g., vegetarian or vegan, low FODMAP, or other empirical exclusion diets) were found, which could represent potentially confounding factors in the evaluation of IBS symptoms. Less than half of the patients showed comorbidities, mainly represented by cardiovascular diseases, dyslipidemia, diabetes, and thyroid diseases. Table 2 shows the demographic and clinical characteristics of the eligible IBS patients who took part in the survey about CAM therapy for their condition. 

### 3.2. CAM Utilization

Table 3 displays the characteristics of patients associated with CAM use. Among the 137 patients included in the final analysis, 62 (45%) described themselves as CAM users, while 75 (55%) denied any use of CAM therapies for IBS (*p* = 0.11). At univariate analysis, CAM use was significantly associated with a higher BMI (*p* = 0.004), being overweight (*p* = 0.02), having a higher level of education (*p* = 0.0006), and having a good level of knowledge about CAM therapies (*p* = 0.0004). Although not reaching statistical significance (*p* > 0.05), having an older age, being female, and not being a smoker showed a trend toward an association with CAM use. Nevertheless, mutual adjustment of variables with each other showed that only being female (*p* = 0.01), having a higher BMI (*p* = 0.02), and having a good level of knowledge about CAM therapies (*p* = 0.002) were independent risk factors for CAM use, while having children represented a protective factor (*p* = 0.04). No significant association was found between CAM use and the other variables, such as IBS type, civil and occupational status, or trust in conventional medicine.

Since gender was found to be an independent factor associated with the use of CAM, we analyzed males and females separately (Table 4 and Table 5). At the univariate analysis, having a higher BMI and having a good level of CAM knowledge still showed a significant association with CAM use, both in men (*p* = 0.02 and *p* = 0.001, respectively) and in women (*p* = 0.03 and *p* = 0.02, respectively). However, multivariate logistic regression analysis showed a higher prevalence of CAM use in those men with less comorbidity (*p* = 0.04) and having a good level of CAM knowledge (*p* = 0.01). In women, higher BMI (*p* = 0.02) and a good level of CAM knowledge (*p* = 0.03) were independently associated with use of CAM, while having children was a protective factor (*p* = 0.008).

### 3.3. Approaches, Motivations, and Preferences among CAM Users

**Figure 1:**
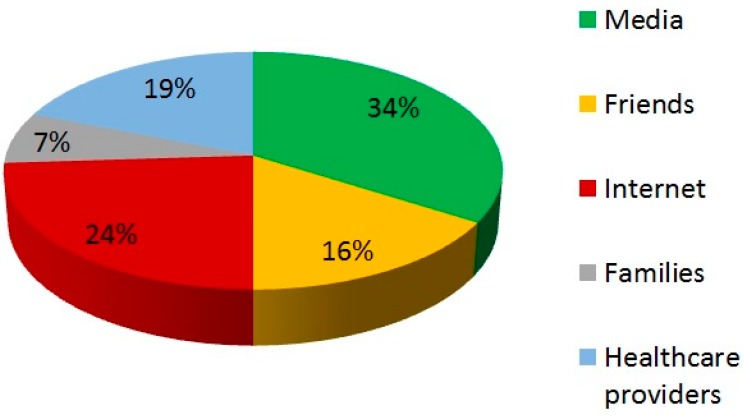
Sources of information among CAM users.

When interviewed regarding the source of information taken into consideration in order to learn more about CAM therapies, more than half (58%) of the patients surprisingly affirmed that they searched for news mostly outside the medical environment. Therefore, it was prominent that media, such as television, radio, and newspapers, were preferred as a source of information than medical advice. Figure 1 summarizes the information sources considered by CAM users.

**Figure 2:**
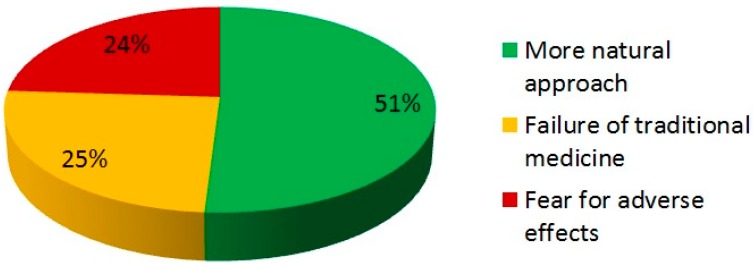
Reasons for choosing CAM therapies.

When CAM users were asked to indicate the most prevalent reason why they chose CAM, it was noted that over half of patients thought it was a “more natural” way to treat gastrointestinal symptoms, while the remaining patients were motivated for the alternative approach because of the lack of therapeutic options offered by traditional medicine or because they expressed apprehension toward adverse drug reactions (Figure 2).

**Figure 3:**
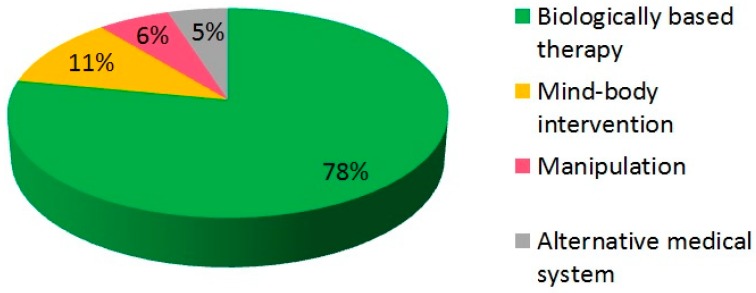
Preferred CAM therapies by participants in the survey.

Natural remedies, such as nutraceuticals and herbal compounds, resulted as the most commonly used CAM. Indeed, 37% of patients reported the use of herbs, 26% the use of nutraceutical compounds, and 15% the use of vitamins, which are all classified as biologically based therapies. Meditation was preferred by 6 (10%) patients, while one woman (1%) reported that art, which belongs to the mind–body intervention category as well as meditation, was the main CAM she used to treat her gastrointestinal symptoms. Among manipulation therapies listed, including massage, chiropractic, and acupuncture, the only type chosen was massage. Similarly, the unique type of alternative medical system appreciated for its results, among those listed in the questionnaire, was homeopathy. These results are summarized in Figure 3.

It is interesting to note that, in our series of patients, none had ever used energy therapies, including reiki, electromagnetic fields, qi gong, and therapeutic touch.

### 3.4. Perceived Benefits from CAM Therapies

**Figure 4:**
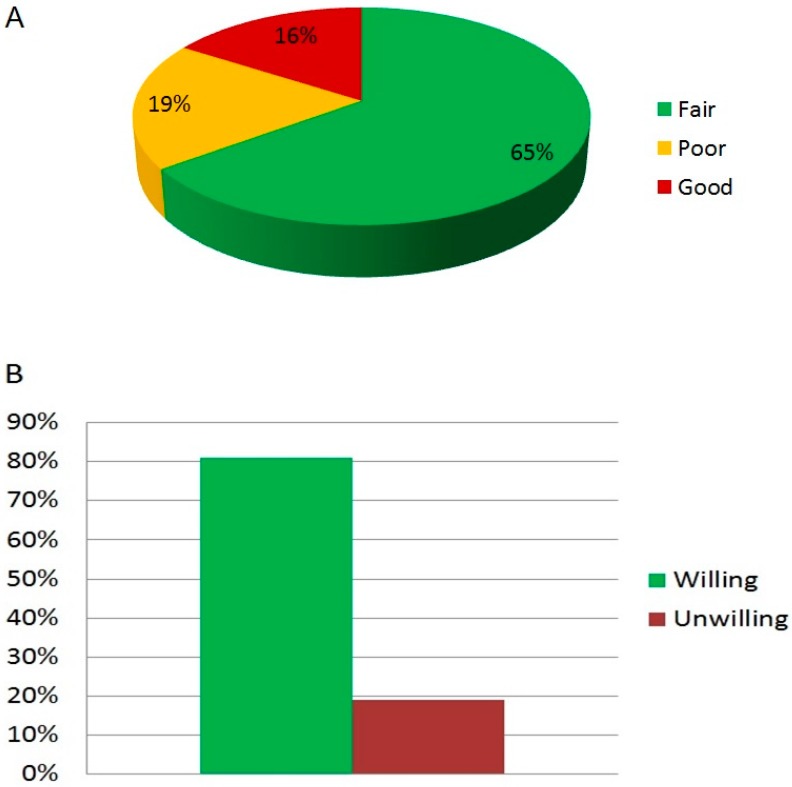
Satisfaction perceived from patients using CAM for IBS (**A**) and their intention to repeat CAM therapy experience for treating IBS (**B**).

When patients were asked whether they were satisfied with the use of CAM, only a minority reported full satisfaction, while more than half of the patients indicated an average benefit after their experience (Figure 4A). However, despite this apparent poor outcome of CAM efficacy, patients were well disposed to re-use CAM for the treatment of their IBS in the future (Figure 4B).

## 4. Discussion

This survey evaluated the spread of CAM use for IBS, as assessed by the Rome IV criteria, in native patients living in the Calabria region of Southern Italy. CAM consists of a wide range of healthcare practices that are not part of traditional medicine and are not integrated into the dominant healthcare system [8]. The therapeutic approaches and the preferences of the persons vary according to their socio-cultural, historical, and sometimes religious values, but CAM use is growing rapidly worldwide, especially among those patients with chronic conditions such as IBS [17]. The only data with regard to CAM experience in IBS Italian patients came from a multicentric survey focusing on pharmacological and non-pharmacological treatments for functional gastrointestinal disorders (FGIDs), which showed that most patients used conventional drugs to relieve symptoms, followed by dietary modifications and CAM (e.g., 81%, 64%, and 49%, respectively) [18]. 

A distinctive aspect of this study has been the consideration of the recent Roma IV criteria for including patients in the survey. In doing so, among the patients who consecutively came to our notice having a previous diagnosis of IBS, 12% did not receive confirmation of the previous diagnosis. This discrepancy, mainly due to the removal of the “discomfort” symptom by the new criteria, is in line with the current, even still limited, data on the topic. A recent Chinese survey conducted on 1376 unselected patients in gastroenterology units concluded that patients with IBS according to the Roma IV criteria represent somehow a subset of patients diagnosed with the Rome III criteria. Indeed, IBS prevalence in the population surveyed was 12% using the Rome III criteria but dropped to 6% when the evaluation was carried out by applying the Rome IV criteria [19]. The latter represent a reference diagnostic breakthrough in scientific research and FGID management. In particular, the Roma IV questionnaire has been translated into many languages and is currently being validated in various countries, in order to overcome the different linguistic interpretations that are potentially confounding factors in epidemiological and clinical evaluations [20,21].

The use of CAM observed in our population was 45.3%, and this is consistent with the current available data. Accordingly, the prevalence of CAM use in the general population in the US between 1997 and 2002 was around 35% and, in particular, this figure was confirmed for the specific treatment of FGIDs [22,23].

Previous studies have found an association between the use of CAM and female gender, higher levels of education, and the presence of comorbidity, both in general and in the treatment of FGIDs [24,25]. This study confirmed that women were more likely than men (73% vs. 27%) to choose CAM to treat IBS.

While a higher BMI has been shown to be an independent risk factor for CAM use, being overweight no longer had a significant association with CAM use after logistic regression analysis was performed. Data on the relationship between CAM experience and body weight are controversial and still being updated [26]. In patients suffering from musculoskeletal disorders, the approach with CAM was more frequent in overweight vs. normal weight subjects, but the same association was not found when considering obese vs. normal weight patients [27]. Similarly, overweight patients with previous malignancies showed a more pronounced inclination to experience CAM than those who were underweight, normal weight, or obese [28]. A likely explanation for this finding might be that some CAM such as acupuncture, yoga, chiropractic, or massages could be less appealing for obese people, creating feelings of self-defense, discomfort, and embarrassment [29].

The finding that IBS patients with children approached CAM to a lesser extent than those without children needs some clarification. Indeed, when a separate analysis has been performed, having children was independently associated with a reduced risk for CAM use only in women, while the association was not observed among men.

Even if it has been reported that single patients were more likely to use CAM than married or cohabiting patients [25,30], the data of this study suggest that is not due to the single status but to the absence of children, which often accompanies the single status. Since children are generally looked after by women more than men, it seems that having children may constitute a barrier for women to pursue CAM use. In recent years, there has been a growing interest in CAM and it is no surprise that a good knowledge of these therapies was significantly higher in CAM users (46%) compared to those who did not use it (18%). This confirms that knowledge of CAM is fundamental for their estimation, as reported by Saha et al. who found that a poor level of knowledge about CAM was the main reason for discontinuing its use, in pharmacy college students [31].

Others found an association between a higher level of education and CAM [23]. In this study, a higher level of education possibly acted as a surrogate of a good CAM knowledge at univariate analysis; however, it was no longer associated with greater CAM use when multivariate analysis was performed.

Trust in conventional medicine did not affect the use of CAM in our population. The relationship between trust in regular health care professionals and consulting alternative practitioners is an intriguing topic, and it would be interesting to predict CAM use by trust in conventional medicine. Some authors reported that a lack of trust in traditional heath care can induce patients to look for alternative remedies [32,33]. However, results obtained after logistic regression analysis in chronically ill people in Netherlands showed no relationship between trust in present health care and the use of CAM [34]. Attitudes toward CAM are often influenced by comparisons within families and friends, while healthcare professionals are less often involved in choices [35]. This data did not quite emerge from this study, where most of the information about CAM came through the media and the web, and only a smaller percentage of subjects relied on the advice of family and friends. According to the current knowledge, the poor disposition of patients to contact their physician in searching for CAM information has been confirmed here. It is probably due to the perception of the scarce trust placed by the healthcare providers toward CAM therapies, or because patients are afraid of seemingly unrealistic claims [36]. A further reason could be due to poor communication between the physician and patient during the clinical visit, which is usually limited to a simple description of the symptoms and advice on traditional therapies [37]. In this regard, it is important to point out how future approaches should be aimed at awareness-raising campaigns for CAM consumers, limiting their use without consulting a health professional or by regulating them adequately [38].

The attitude for CAM use is mainly due to the feeling of a natural approach to treatment of illnesses, but this perception of a lack of side effects is often different from reality. Indeed, despite the good safety profile shown by CAM [39], it still represents a therapeutic intervention and, as such, potentially burdened by adverse events, whether it is going to treat IBS, a relatively benign pathologies, or it is employed in cancer [40,41]. Accordingly, in this survey the more natural approach was the main motivation (51%) that drives patients to introduce CAM into their daily strategies to counteract IBS. In a Turkish study questioning patients with IBS as to the benefits that could be expected from CAM, 31.5% of patients stated that it was useful in improving psycho-physical well-being in general, while 27% replied that CAM “cannot hurt“ [14].

The consumption of nutraceutical compounds and herbal preparations was the most practiced by participants in this survey, according to current literature [42]. On the other hand, a small number of patients placed meditation and massages first, and no patient had ever considered energy therapies; those results are in line with other surveys in IBS population [43,44,45].

After questioning about their satisfaction level derived from CAM, only a minor percentage (16%) of the respondents fully promoted the experience, and indeed little benefits among CAM users have already been reported. Kav et al. assessed the positive judgment on CAM on a visual scale of 1–7, with higher scores indicating a higher level of satisfaction. The mean value, considered as the overall efficacy of the CAM tested, was 3.86 ± 2.3 [14]. Surprisingly, 81% of our participants stated their willingness to turn to a complementary treatment for IBS in the future. This apparent contradiction indicates how much effort is required from the researchers and clinicians, to better define the factors involved in CAM therapies, which are responsible for the wide variability of results obtained in different settings.

Limitations of the present study could be represented by the small number of patients and the single-center conduction. However, it must be emphasized that the enrolled patients were all local residents, representing a quite homogeneous community-based sample, and therefore limiting the influence of various cultural factors.

## 5. Conclusions

In a native local resident setting of IBS patients from Southern Italy, classified according to the Rome IV criteria, nearly half of them were involved in the use of CAM. A healthcare providers’ approach must take into account the possibility that patients use CAM therapies. Therefore, CAM preference should be investigated as part of the medical interview and discussed with patients impartially. Attention to CAM and to the patient’s motivations for their employment is a fundamental issue for health professionals, who must consider the use of a carefully planned clinical interview as well as the improvement of diagnostic techniques and the development of innovative therapeutic strategies.

## Figures and Tables

**Table 1:** Rome IV criteria [13].

Recurrent abdominal pain, on average, at least 1 day per week in the last 3 months, associated with 2 or more of following criteria *:
1. Related to defecation 2. Associated with a change in frequency of stool 3. Associated with change in form (appearance) of stool

* Criteria fulfilled for the last 3 months with symptom onset at least 6 months before diagnosis.

**Table 2:** Demographic and clinical characteristics of the 137 irritable bowel syndrome (IBS) patients as assessed by Roma IV criteria.

Characteristics	Males *n* = 49	Females *n* = 88	Total *n* = 137
Age, median (range), years	53 (18–83)	38 (18–84)	42 (18–84)
BMI, mean ± SD, kg/m^2^	25.7 ± 3.2	23.7 ± 4.5	24.4 ± 4.1
Overweight, *n* (%)	28 (57)	22 (25)	50 (36)
Working status			
Employee, *n* (%)	29 (59)	5 (10)	63 (46)
Retired, *n* (%)	10 (20)	13 (15)	23 (18)
Housewife, *n* (%)	0 (0)	14 (16)	14 (10)
Unemployed, *n* (%)	5 (10)	7 (8)	12 (8)
Student, *n* (%)	5 (10)	20 (23)	25 (18)
High education, *n* (%)	31 (63)	61 (69)	92 (67)
Smoking, *n* (%)	17 (35)	12 (14)	29 (21)
Anxiety/depression, *n* (%)	8 (16)	11 (13)	19 (14)
Comorbidity, *n* (%)	22 (45)	33 (37)	55 (40)
Drugs for comorbidity, *n* (%)	15 (30)	27 (30)	42 (30)
Married/Cohabiting, *n* (%)	33 (67)	56 (64)	81 (59)
Having children, *n* (%)	29 (59)	42 (48)	71 (52)
Previous major surgery, *n* (%)	33 (67)	56 (64)	89 (65)
Family history of cancer, *n* (%)	24 (49)	43 (49)	67 (49)
Trust in conventional medicine, *n* (%)	28 (57)	58 (65)	89 (65)
Good CAM knowledge, *n* (%)	17 (34)	26 (29)	43 (31)

BMI = body mass index; SD = standard deviation; values are numbers (%), mean ± SD or median with range, as indicated.

**Table 3:** Characteristics of the 137 patients with IBS according to complementary and alternative medicine (CAM) status as assessed by questionnaire.

Variables	Cam Users *n* = 62	Non Cam Users *n* = 75	*p*	OR (95% CI) Adjusted ^a^	*p*
Sex, *n* (%)					
Male	17 (27)	32 (43)	0.06	7.22 (2.31–22.51)	**0.01**
Female	45 (73)	43 (57)			
Age, median (range), years	52 (19–82)	38 (19–82)	0.05	1.04 (0.98–1.10)	0.13
BMI, mean ± SD, kg/m^2^	25.4 ± 3.8	23.7 ± 4.5	**0.00**	1.16 (1.02–1.33)	**0.02**
Overweight, *n* (%)	29 (47)	21 (28)	**0.02**	2.56 (0.82–7.97)	0.10
Smoking, *n* (%)	9 (14)	20 (27)	0.08	1.17 (0.36–3.79)	0.79
IBS type *n* (%)					
C	24 (39)	20 (27)	0.13	2.54 (0.87–7.43)	0.08
D	11 (18)	19 (25)	0.28	1.11 (0.34–3.62)	0.85
U	7 (11)	6 (8)	0.51	3.92 (0.70–21.81)	0.11
M ^b^	20 (32)	30 (40)	0.34	------------------	------
High education, *n* (%)	51 (82)	41 (54)	**0.00**	1.05 (0.36–3.01)	0.92
Marriage/Cohabiting, *n* (%)	36 (58)	45 (60)	0.8	0.79 (0.21–2.97)	0.73
Current Worker, *n* (%)	27 (43)	36 (48)	0.6	1.22 (0.51–2.91)	0.63
Anxiety/Depression, *n* (%)	8 (13)	11 (15)	0.8	0.96 (0.27–3.38)	0.95
Comorbidity, *n* (%)	26 (41)	29 (38)	0.4	0.35 (0.06–1.97)	0.23
Drugs for comorbidity, *n* (%)	20 (32)	22 (29)	0.6	1.80 (0.33–9.66)	0.49
Having children, *n* (%)	31 (50)	40 (53)	0.7	0.25 (0.07–0.95)	**0.04**
Previous major surgery, *n* (%)	41 (66)	48 (64)	0.8	0.74 (0.27–2.03)	0.56
Familiarity for cancer, *n* (%)	29 (46)	38 (50)	0.7	0.65 (0.26–1.58)	0.34
Trust in conventional medicine, *n* (%)	40 (64)	49 (65)	0.9	1.06 (0.42–2.68)	0.88
Good CAM knowledge, *n* (%)	29 (46)	14 (18)	**0.00**	4.46 (1.73–11.45)	**0.002**

BMI = body mass index; CI = confidence interval; MVA = multivariate analysis; OR = odds ratio; SD = standard deviation; values are numbers (%), mean ± SD or median with range as indicated; means were compared with the use of a Student’s *t*-test when data were normally distributed and with a Mann–Whitney U-test when data were not normally distributed, and proportions were determined with the use of a chi-square test. ORs with 95% CI in brackets are given. Bold text indicates a statistically significant difference with a *p* value less than 0.05; ^a^ All variables except age and BMI entered MVA analysis as categorical variables. ^b^ This parameter is set to zero because it is redundant.

**Table 4:** Characteristics of the 49 male patients with IBS according to CAM status as assessed by questionnaire.

Variables	Cam Users *n* = 17	Non Cam Users *n* = 32	*p*	OR (95% CI) Adjusted ^a^	*p*	Total *n* = 49
Age, median (range), years	63 (19–82)	46.5 (18–83)	0.10	0.99 (0.89–1.09)	0.85	53 (18–83)
BMI, mean ± SD, kg/m^2^	26.9 ± 2.8	24.9 ± 3.0	**0.02**	1.36 (0.84–2.19)	0.20	25.6 ± 3.0
Overweight, *n* (%)	14 (82)	14 (43)	**0.00**	1.82 (0.20–15.67)	0.13	28 (57)
Smoke, *n* (%)	3 (17)	12 (37)	0.15	1.08 (0.10–10.83)	0.94	15 (30)
IBS type, *n* (%)						
C	9 (52)	11 (34)	0.20	0.31 (0.02–4.38)	0.39	20 (40)
D	2 (11)	4 (12)	0.94	3.36 (0.08–137.89)	0.52	6 (12)
U	4 (23)	5 (15)	0.49	0.04 (0.002–1.27)	0.06	9 (18)
M ^b^	2 (11)	12 (37)	0.05	------------------	------	14 (28)
High education, *n* (%)	10 (58)	21 (65)	0.63	1.90 (0.22–15.96)	0.55	31 (63)
Marriage/Cohabiting, *n* (%)	12 (70)	20 (62)	0.57	0.23 (0.004–13.00)	0.47	32 (65)
Current Worker, *n* (%)	10 (58)	20 (62)	0.80	1.41 (0.22–9.11)	0.71	30 (61)
Anxiety/Depression, *n* (%)	3 (17)	4 (12)	0.62	3.19 (0.20–50.25)	0.40	7 (14)
Comorbidity, *n* (%)	7 (41)	15 (46)	0.70	36.6 (1.24–108.3)	**0.04**	22 (44)
Drugs for comorbidity, *n* (%)	6 (35)	9 (28)	0.60	0.034 (0.001–1.52)	0.08	15 (30)
Having children, *n* (%)	12 (70)	17 (53)	0.23	0.89 (0.03–20.20)	0.94	29 (59)
Previous major surgery, *n* (%)	11 (64)	21 (65)	0.94	5.54 (0.24–126.58)	0.28	32 (65)
Familiarity for cancer, *n* (%)	9 (52)	15 (46)	0.68	0.19 (0.01–2.52)	0.20	24 (48)
Trust in conventional medicine, *n* (%)	7 (41)	20 (25)	0.15	11.35 (0.08–59.58)	0.07	27 (55)
Good CAM knowledge, *n* (%)	11 (64)	6 (18)	**0.00**	14.36 (2.62–78.74)	**0.01**	17 (34)

BMI = body mass index; CI = confidence interval; MVA = multivariate analysis; OR = odds ratio; SD = standard deviation; values are numbers (%), mean ± SD or median with range as indicated; means were compared with the use of a Student’s *t*-test when data were normally distributed and a Mann–Whitney U-test when data were not normally distributed, and proportions were determined with the use of a chi-square test. ORs with 95% CI in brackets are given. Bold text indicates a statistically significant difference with a *p*-value less than 0.05; ^a^ All variables except age and BMI entered MVA analysis as categorical variables. ^b^ This parameter is set to zero because it is redundant.

**Table 5:** Characteristics of the 88 female patients with IBS according to CAM status as assessed by questionnaire.

Variables	Cam Users *n* = 45	Non Cam Users *n* = 43	*p*	OR (95% CI) Adjusted ^a^	*p*	Total *n* = 49
Age, median (range), years	41(19–79)	37 (18–84)	0.21	1.04 (0.98–1.10)	0.28	38 (18–84)
BMI, mean ± SD, kg/m^2^	24.7 ± 3.8	22.7 ± 4.8	**0.03**	1.20 (1.02–1.42)	**0.02**	23.7 ± 4.4
Overweight. *n* (%)	15 (33)	7(16)	0.06	2.07 (0.48–8.98)	0.32	22 (25)
Smoking, *n* (%)	6 (13)	6 (13)	0.93	1.93 (0.37–10.07)	0.43	12 (13)
IBS type, *n* (%)						
C	15 (33)	9 (20)	0.19	2.50 (0.70–8.97)	0.15	24 (27)
D	9 (20)	15 (33)	0.11	1.13 (0.30–4.25)	0.85	24 (27)
U	3 (6)	1 (2)	0.32	16.08 (0.41–630.70)	0.13	4 (4)
M ^b^	18 (40)	18 (41)	0.85	------------------	------	36 (40)
High education, *n* (%)	31 (68)	29 (67)	0.88	1.17 (0.30–4.51)	0.81	60 (68)
Marriage/Cohabiting, *n* (%)	24 (53)	24 (55)	0.81	0.77 (0.12–4.72)	0.78	48 (54)
Current Worker, *n* (%)	19 (42)	16 (37)	0.63	1.86 (0.58–5.93)	0.28	35 (39)
Anxiety/Depression, *n* (%)	5 (11)	6 (13)	0.68	0.83 (0.18–3.83)	0.81	11 (12)
Comorbidity, *n* (%)	19 (42)	14 (32)	0.34	0.45 (0.03–5.19)	0.52	33 (37)
Drugs for comorbidity, *n* (%)	16 (35)	11 (25)	0.31	2.12 (0.14–31.88)	0.58	27 (30)
Having children, *n* (%)	19 (42)	23 (53)	0.29	0.09 (0.01–0.54)	**0.00**	42 (47)
Previous major surgery, *n* (%)	30 (64)	26 (65)	0.54	5.54 (0.24–126.58)	0.28	56 (63)
Familiarity for cancer, *n* (%)	20 (44)	23 (53)	0.39	0.64 (0.21–1.91)	0.42	53 (60)
Trust in conventional medicine, *n* (%)	33 (73)	29 (67)	0.54	2.01 (0.60–6.76)	0.25	62 (70)
Good CAM knowledge, *n* (%)	18 (40)	8 (18)	**0.02**	3.65 (1.10–12.15)	**0.03**	26 (29)

BMI = body mass index; CI = confidence interval; MVA = multivariate analysis; OR = odds ratio; SD = standard deviation; values are numbers (%), means ± SD or median with range, as indicated; means were compared with the use of a Student’s t-test when data were normally distributed and a Mann–Whitney U-test when data were not normally distributed, and proportions were determined with the use of a chi-square test. ORs with 95% CI in brackets are given. Bold text indicates a statistically significant difference with a p-value less than 0.05; ^a^ All variables except age and BMI entered MVA analysis as categorical variables. ^b^ This parameter is set to zero because it is redundant.

## Supplementary Materials

The following are available online at http://www.mdpi.com/1010-660X/55/2/46/s1.
