# Supplementary material for: Use of Complementary and Alternative Medicine by Patients with Irritable Bowel Syndrome According to the Roma IV Criteria: A Single-Center Italian Survey
Source: Medicina (Kaunas). 2019 Feb 13;55(2):46. doi: 10.3390/medicina55020046 (PMC6409648; doi:10.3390/medicina55020046)
Supplement: Supplementary file 1 [file medicina-55-00046-s001.pdf]

Questionnaire for Survey of Complementary and Alternative Medicine (CAM) use in Irritable Bowel Syndrome (IBS)

1) Among the following, which CAM you most frequently used for IBS in the last 12 months:
☐ Homeopathy ☐ Chinese Medicine ☐ Ayurvedic Medicine ☐ Meditation ☐ Prayer ☐ Mental Training ☐ Art Therapy ☐ Herbs ☐ Nutraceuticals ☐ Vitamins
☐ Manipulation Therapies ☐ Chiropractic ☐ Massage ☐ Healing Touch
☐ Qi Gong ☐ Reki ☐ Electromagnetic fields

2) How would you judge your degree of trust in traditional medicine?
☐ Poor ☐ Fair ☐ Good

3) What is your degree of knowledge of unconventional medicine?
☐ Poor ☐ Fair ☐ Good

4) How do you consider the intervention with CAM for your IBS?
☐ Poor ☐ Fair ☐ Good

5) Will you repeat CAM use for IBS in the future?
☐ Yes ☐ No

6) Who gave you information about CAM?
☐ Media (television, newspapers) ☐ Healthcare providers ☐ Family ☐ Internet ☐ Friends

7) Why did you chose CAM for your IBS?
☐ Fear from conventional drugs adverse reaction
☐ Feeling of a ''more natural'' approach
☐ Failure of conventional therapy
